# Modified Habitats Influence Kelp Epibiota via Direct and Indirect Effects

**DOI:** 10.1371/journal.pone.0021936

**Published:** 2011-07-06

**Authors:** Ezequiel M. Marzinelli, Antony J. Underwood, Ross A. Coleman

**Affiliations:** Centre for Research on Ecological Impacts of Coastal Cities, School of Biological Sciences, University of Sydney, Sydney, New South Wales, Australia; Institute of Marine Research, Norway

## Abstract

Addition of man-made structures alters abiotic and biotic characteristics of natural habitats, which can influence abundances of biota directly and/or indirectly, by altering the ecology of competitors or predators. Marine epibiota in modified habitats were used to test hypotheses to distinguish between direct and indirect processes. In Sydney Harbour, kelps on pier-pilings supported greater covers of bryozoans, particularly of the non-indigenous species *Membranipora membranacea*, than found on natural reefs. Pilings influenced these patterns and processes directly due to the provision of shade and indirectly by altering abundances of sea-urchins which, in turn, affected covers of bryozoans. Indirect effects were more important than direct effects. This indicates that artificial structures affect organisms living on secondary substrata in complex ways, altering the biodiversity and indirectly affecting abundances of epibiota. Understanding how these components of habitats affect ecological processes is necessary to allow sensible prediction of the effects of modifying habitats on the ecology of organisms.

## Introduction

Anthropogenic modification of habitats (e.g. land-use for agriculture, urbanization) is probably the primary cause of the current loss of biodiversity because it influences and interacts with other anthropogenic disturbances [1], [2]. Identifying the processes by which modified habitats affect biodiversity is, therefore, necessary to mitigate human impacts.

Modified habitats change abiotic and biotic characteristics, influencing the distribution, diversity and abundances of organisms [3]. When multiple components of habitat are simultaneously modified, any differences in the assemblages of organisms may be due to different abiotic characteristics of the habitat or due to modifications of biological interactions among organisms caused by the alteration of the habitat [1], [4]. Roadsides, for example, differ from surrounding natural habitats in several abiotic characteristics [5], [6] that can directly influence covers of invasive plants [7]. Alternatively, effects of roads on invasive plants can be indirect, i.e. roads change the ecology or behaviour of organisms, which, in turn, affect abundances of plants. An example of this is the dispersal of seeds of invasive plants by birds. Abundances of birds are greater on roadsides because these provide sites for nesting and roosting [8], [9]. Birds then feed on fruits of invasive plants and disperse the seeds along roadsides, which results in increases in abundances of these plants [9]. Roadsides may thus have direct and/or indirect effects.

It is very difficult to separate potential direct and indirect effects when abiotic and biotic components of habitat are simultaneously modified, as these effects are inherently confounded. For instance, modification of habitats may influence abundances of organisms indirectly by altering some aspect of the behaviour or ecology of competitors or predators (as with birds on roads). Manipulative experiments are therefore necessary to unconfound such factors and to provide better mechanistic understanding to mitigate impacts on natural systems. Epibiota on kelps in modified habitats were used here in experiments to unconfound and determine the relative importance of direct *vs* indirect effects of human modification of habitat.

One of the effects of urbanization in coastal cities is the modification of natural habitats by the addition of artificial (i.e. man-made) structures for commercial and recreational purposes. These artificial structures are intrinsically different from natural habitats, e.g. they often have vertical surfaces (e.g. seawalls and pilings), are made from different substrata (e.g. wood, concrete) and cause shade (e.g. piers) [10], [11]. These structures support assemblages different from those in many natural habitats [12], [13], [14]. Assemblages on biogenic habitats on artificial structures also differ from those in natural habitats [15], [16]. Influences of modified habitats are therefore relevant for epibiota on secondary substrata, such as kelps or other biogenic habitats.

The effects of modification of habitat on epibiota may have serious consequences for other organisms, thereby affecting the functioning of these systems. In Sydney Harbour, the kelp *Ecklonia radiata* occurs on artificial structures and on natural reefs, providing habitat for many organisms, amongst which bryozoans are common and abundant. In particular, abundances of the non-indigenous bryozoan *Membranipora membranacea* [17] are significantly greater on kelps on pier-pilings than on kelps on natural reefs. This species covers most of the laminae of the kelps occurring on pilings [15], which can have negative effects on the kelps (e.g. reduced area for photosynthesis and gas-exchange) [18], [19]. This can contribute to the loss of stands of kelps, which provide habitat and other resources to a wide variety of organisms [20], [21].

Because bryozoans do not occur directly on pilings, influences of these structures can be kelp-mediated, i.e. pier-pilings change characteristics of the kelps, which, in turn, affect abundances of bryozoans. Experimental transplants of kelps, however, showed that greater covers of bryozoans on kelps on pilings were caused by differences in abiotic factors and/or biological interactions between modified and natural habitats (primary habitats). They were not due to properties of the kelp itself (secondary habitat) [15].

One possible explanation for the difference in abundances of bryozoans on kelps is differences in abiotic factors between modified and natural habitats. Piers often shade organisms on the pilings. Kelps are shaded to different extents on pilings compared with rocky reefs. Shade has positive effects on recruitment of many organisms [22] and may thus increase settlers of bryozoans on kelps on pilings. Alternatively, bryozoans may be affected by differences in biological interactions between habitats. For instance, modification of habitat may influence abundances of competitors or predators that may, in turn, affect covers of bryozoans. In forests of *E. radiata* in Sydney, the sea-urchin *Holopneustes purpurascens* occurs in great abundances, reaching densities of up to 1 individual per kelp and greater than 17 per m^2^ [23]. Abundances of *H. purpurascens* appeared to be much greater in forests of *E. radiata* on rocky reefs than on pilings. Because urchins feed on bryozoans and on laminae of the kelps they inhabit [23], [24], [25], differences in abundances of urchins between habitats may explain the observed patterns of abundances of bryozoans. Urchins may, however, affect bryozoans in other ways besides consumption [26], [27]. Independently of the mechanism, smaller abundances of urchins on pilings could thus cause greater covers of bryozoans on kelps. The effect of modification of habitat on covers of bryozoans would then be indirect.

Preliminary experiments examining the effects of shade and urchins independently, suggested that both factors influenced covers of bryozoans [28]. Examining these factors independently did not allow, however, determining the relative importance of direct *vs* indirect effects or their interactive effects. The purpose of this study was therefore to unconfound and quantify the relative importance of direct and indirect processes mediating the impact of modification of habitat on covers of invasive bryozoans. We examined the models that greater covers of bryozoans on kelps on pilings are caused: (1) directly, by greater shade on pilings; (2) indirectly, by smaller predation/ disturbance by urchins on pilings; or (3) by a combination of both. First, we quantified densities of urchins to test the hypothesis that urchins occur in greater densities on kelps on reefs than on those on pilings. Model 1 leads to the prediction that on experimentally shaded kelps on reefs, covers of bryozoans will increase to match covers on kelps on pilings. Model 2 leads to the predictions that (a) on reefs from which urchins have been experimentally excluded, covers of bryozoans will increase to match covers on kelps on pilings; (b) on pilings where urchins have been experimentally added, covers of bryozoans will decrease to match those on kelps on reefs. Finally, model 3 leads to the prediction of an interaction between exclusion of urchins and shade; i.e. covers of bryozoans will be greater on shaded kelps on reefs from which urchins have been experimentally excluded in comparison to unshaded kelps or those with urchins.

## Material and Methods

### Study Location

Experiments were done at Balmoral Beach (BB; 33°49′S 151°15′E; NSW Fisheries research permit F96/146-6.0) in Sydney Harbour, NSW, Australia. BB is approximately 2 km apart near the entrance of the harbour and has relatively little exposure to waves. Artificial structures in this location include public swimming-pools surrounded by shark-nets and wharves with wooden pilings and decking, all built over soft sediment at depths of approximately 2–6 m. All pilings had attached epibiota, including foliose algae (predominantly *E. radiata*), filamentous algae, bryozoans, polychaetes, sponges, ascidians, hydroids, barnacles and oysters. *E. radiata* growing on pilings were at approximately the same depth (0–3 m) as on rocky reefs. Natural rocky reefs in these locations are extensive sandstone platforms with crevices and patches of *E. radiata* at depths of 0–3 m. The epibiota on reefs are similar to those on pilings, but more patchily distributed. The minimal distance between reefs and pilings was approximately 350 m.

### Abundances of urchins

Before the experiment was done, densities of *H. purpurascens* (>5 cm diameter of test) were measured on rocky reefs by haphazardly placing a 1 m^2^ quadrat every 10 m along a 100 m transect parallel to and 10 m from the shore. Urchins were counted on 10 randomly-selected pilings along a transect in the direction of the pier. Within the 1 m^2^ quadrat on reefs or on pilings, one individual *E. radiata* was haphazardly chosen and the numbers of urchins per kelp were recorded.

### Experimental design

The effects of shade and urchins on covers of bryozoans were investigated simultaneously by an orthogonal experiment with both factors at the reefs in BB. Thirty-six patches of 1.5×1.5 m were selected haphazardly at the same depth (∼1–2 m) and marked with flagging tape. Four patches of *E. radiata* were randomly assigned to each of 9 combinations of shading and urchin removal treatments: i) Shade Excluded (SE) – kelps were shaded using a shading structure (see *Material and methods: Shade*) and all encountered individual *H. purpurescens* were removed from the patch; ii) Shade Disturbed (SD) – kelps were shaded and urchins were disturbed as required by the exclusion treatment, but were returned to their original patch; iii) Shade Undisturbed (SU) – kelps were shaded and no manipulation of urchins was done; iv) Control Excluded (CE) – kelps under a similar structure, but which did not shade (see *Material and methods: Shade*) and all encountered individual *H. purpurescens* were removed; v) Control Disturbed (CD) – kelps under a control structure and urchins were disturbed as required by the exclusion treatment, but were returned to their original patch; vi) Control Undisturbed (CU) – kelps under a control structure and no manipulation of urchins was done; vii) Exclusion (E) – all encountered *H. purpurescens* were removed from the patch; viii) Disturbed (D) - urchins were disturbed as required by the exclusion treatment, but were returned to their original patch; ix) Undisturbed reefs (U_r_) – no manipulation was done. Undisturbed kelps on pilings (P) were also sampled to allow comparison of covers of bryozoans on kelps under the 9 treatments on reefs to those occurring on pilings. Eight individuals of *E. radiata*, each from 8 randomly chosen pilings, were selected haphazardly and marked *in situ* with cable-ties.

### Shade

The shading structures were constructed from tarpaulins of heavy-duty fabric (three-rivet reinforced corners with molded plastic) with grommets around them (∼45 cm apart; Mayo Hardware, China). Each tarpaulin was attached to a frame of PVC pipe (10 cm diameter) bent into a circle (1.5 m diameter) and secured using cable-ties. Shades were held in place by 4 eye-bolts (8 mm) using 4 m long 10 mm polypropylene rope (Ibex, China). Tent-springs (coils) were attached to the eye-bolts and rope to reduce tension. The eye-bolts were drilled into the rock ∼5 m apart in a square. Floats were attached underneath each tarpaulin to prevent them from moving down and disturbing the kelps. Tarpaulins were ∼1 m above the canopy. Control structures were built and attached in the same way, but garden mesh (mesh size: 10×10 cm) was used instead of tarpaulin to allow light to penetrate. The structures were checked every 2 weeks to remove drifting algae that had attached to control structures and might cause shade.

To establish the effectiveness of the shade treatment, the light reaching the canopy of kelps on reefs and the kelps on pilings was measured with a light-sensor of a diving PAM (pulse amplitude modulated; WALZ, Germany). This was done at 5 patches of shade, control and undisturbed treatments and at 2 sites separated by ∼50 m. There was significantly (about 90%) less light on shaded patches or pilings than on control and undisturbed patches on reefs (ANOVA, *F*_3,4_ = 2190, *P*<0.01).

### Exclusion of urchins

Urchins were manually removed by SCUBA divers. All urchins were removed from patches of treatment E to mimic densities on pilings, where essentially no urchins were found (*Results* *: Abundances of urchins*). This procedure was repeated weekly for patches where they were excluded. The number of urchins in each patch was recorded after 2 weeks and 1 month. These time-intervals were chosen because previous observations indicated that urchins return after approximately 15 days to the patches from where they had been excluded.

### Sampling

Two *E. radiata* were haphazardly chosen within each patch and marked *in situ* with cable-ties around the stipe (none of these was at the edge of the patch). At the start of the experiment (5 January 2009) and after 1 month (9 February 2009) each kelp was sampled by taking 5 randomly sited photographs of the primary and secondary laminae. A frame was mounted to the camera to ensure that each image was always the same distance from the substratum (6 cm) and covered the same area (4×5 cm), which provided the greatest possible resolution and precision. Photographs were analysed using the images on a computer screen; percentage covers of bryozoans (individual taxa) were estimated using 30 regularly-spaced points over each photograph. Taxa in a quadrat, but not under these points, were defined to have an arbitrary cover of 0.5%. Animals were identified to the greatest taxonomic resolution possible. The experiment could not last for more than 1 month because storms removed almost all the structures.

### Transplantation of urchins

To test hypothesis *b* of model 2, individual *H. purpurascens* (>5 cm diameter of test) from reefs were experimentally transplanted to *E. radiata* on pilings. Forty urchins were collected manually from the rocky reefs whilst SCUBA diving and placed in a 60 l insulated-plastic container with aerated seawater. The container was then carried to the pilings. Urchins spent only approximately 5 minutes in the container. Two sites separated by ∼50 m were chosen; within each site, 10 pilings were selected haphazardly and assigned randomly to each of 2 treatments: i) Transplanted (TP) – *H. purpurascens* (*n* = 5 to represent the mean density at which they occur on reefs; see *Results* *: Abundances of urchins*) were manually placed on the laminae of randomly-chosen individuals of *E. radiata*; ii) Undisturbed pilings (P) – no urchins were added. The area of each piling covered by kelps was ∼1.5×1.5 m. It was not necessary to cage the urchins because this species wraps itself in the laminae and holds on by its tube-feet. One *E. radiata* was haphazardly chosen within each piling and marked *in situ* with cable-ties around the stipe. The number of urchins in each patch was recorded after 2 weeks and 1 month to account for losses.

Any artefacts of manipulating urchins and transplanting them were examined by adding three treatments [29]: iii) Translocated (TL) – individual *H. purpurascens* were disturbed as necessary to transplant them, but were taken to another site on reefs; iv) Disturbed (D) – individuals were disturbed as necessary to transplant them, but were returned to their original site on reefs; v) Undisturbed reefs (U) – no manipulation was done. These three treatments were compared to determine whether handling and moving the urchins had an effect on the abundances of bryozoans on *E. radiata*. Also, the latter treatment (U) allowed comparison of abundances of bryozoans on laminae of *E. radiata* on pilings in the transplantation treatment with those occurring on reefs.

This experiment was done twice: spring 2006 (3 November to 5 December) and autumn 2007 (25 April to 29 May). At the end of the experiments (1 month), each kelp was sampled as described above (Material and Methods: Sampling).

## Results

### Abundances of urchins

Densities of *H. purpurascens* on reefs ranged between 1–9 per m^2^ (mean, 3.5 ± S.E. 0.4; *n* = 10). Numbers of urchins per individual of *E. radiata* on rocky reefs ranged between 0–5 with a mean of 0.8 ± S.E. 0.5 (*n* = 10). On pilings, only 2 *H. purpurascens* were found, on different kelps on different pilings.

### Shade and exclusion of urchins

At the start of the experiment few kelps sampled on reefs had any bryozoans on their laminae. When present, covers of bryozoans (mainly *M. membranacea* and *B. stolonifera*) were generally small (<5%).

**Figure 1:**
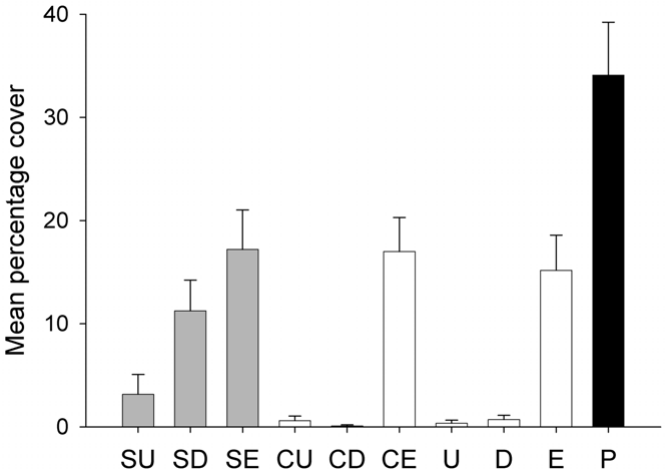
Covers of bryozoans on kelps on reefs after experimental exclusion of urchins and shading. Mean (± S.E.; *n* = 8) percentage cover of bryozoans on kelps 1 month after experimental exclusion of urchins and shading. Treatments are: Shade Undisturbed (SU), Shade Disturbed (SD), Shade Excluded (SE), Control Undisturbed (CU), Control Disturbed (CD), Control Excluded (CE), Undisturbed reefs (U), Disturbed (D), Excluded (E) and Undisturbed Pilings (P).

After 1 month, there was no interaction between the exclusion of urchins and shade (ANOVA, *F*_4,27_ = 0.27, *P*>0.05; Table 1). Covers of bryozoans were greater on kelps on patches from which urchins were excluded than on kelps in control patches (ANOVA, *F*_2,27_ = 5.59, *P*<0.01; Table 1). Despite significant increases in covers of bryozoans on kelps in patches without urchins, covers of bryozoans did not become similar to those on kelps on pilings (Figure 1). Covers of bryozoans were also greater on shaded kelps than on undisturbed kelps on reefs, but this difference was not significant (ANOVA, *F*_2,27_ = 0.69, *P*>0.05; Table 1; Figure 1). No urchins returned to the exclusion patches throughout the experiment. All other patches had between 2–5 adult urchins (Table 2).

**Table 1:** Analysis of mean percentage covers of bryozoans on *E. radiata* for each individual 1 month after the shade and urchin exclusion experiment in 2009.†

Source	*df*	MS	*F*	*P*
Shade S	2	959	0.69	ns
Urchin U	2	7794	5.59	**
S×U	4	380	0.27	ns
Patch P (S×U)	27	1395	9.92	**
Kelp (P (S×U))	36	141	1.14	ns
Residual	288	124		

†Shade was a fixed factor with 3 levels; Urchin was fixed and orthogonal with 3 levels; Patch was a random factor nested in Shade×Urchin with 4 levels and Kelp was random and nested with 2 levels. The replicates were the quadrats (*n* = 5). Cochran's test was significant (*C* = 0.14 **). Transformation of data failed to make variances homogenous, so data were not transformed. **, *P*<0.01; ns, *P*>0.05.

**Table 2:** Mean (± SE; *n* = 4) numbers of sea-urchins per treatment during the experimental exclusion of urchins and shade on reefs in 2009.†

Treatment	SU	SD	SE	CU	CD	CE	U	D	E
No. of urchins	3.5±0.9	3.5±0.6	0	2.5±1.0	2.3±1.3	0	3.8±0.8	2.8±0.8	0

†SU, Shade Undisturbed; SD, Shade Disturbed; SE, Shade Excluded; CU, Control Undisturbed; CD, Control Disturbed; CE, Control Excluded; U, Undisturbed; D, Disturbed; E, Excluded.

Increases in covers of bryozoans were due mainly to *M. membranacea* (covers >90% of total covers of bryozoans). Overall, 80% of covers of bryozoans on kelps on pilings were explained by effects of shade and urchins. Shade explained approximately 30% of covers on pilings, whilst urchins explained approximately 50%.

### Transplantation of urchins

Two species of bryozoans were found on pilings: *M. membranacea* and *B. stolonifera*. Covers of *M. membranacea* represented >90% of total covers of bryozoans. This was consistent in 2006 and 2007.

**Figure 2:**
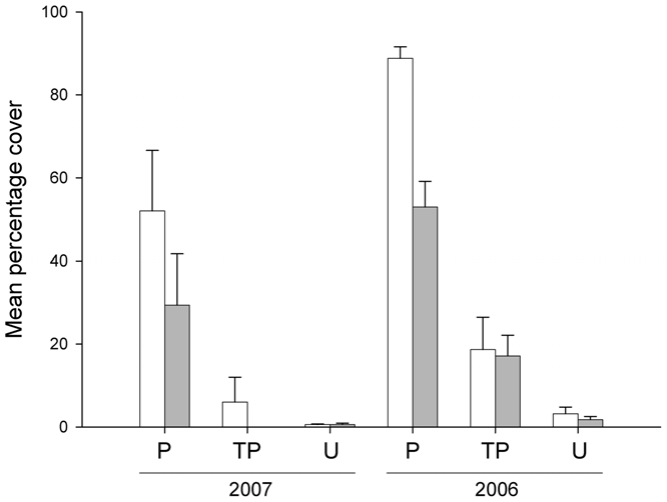
Covers of bryozoans on kelps on pilings after experimental addition of urchins. Mean (± S.E.; *n* = 5) percentage cover of bryozoans on kelps 1 month after urchins were transplanted to pilings in 2006 and 2007. Treatments are: Undisturbed pilings (P), Transplanted (i.e. kelps with sea-urchins; TP) and Undisturbed reefs (U). Site 1, white bars; Site 2, grey bars.

In 2007, covers of bryozoans were significantly smaller on kelps with urchins (TP) than on undisturbed kelps (P; ANOVA, *F*_1,16_ = 14.49, *P*<0.01; Table 3; Figure 2). All urchins transplanted to pilings (*n* = 5) remained during the experiment. No urchins were found on kelps assigned to the undisturbed treatment on pilings. Mean covers of bryozoans on kelps to which urchins had been added (TP) became similar to those on reefs (U) in each site (*t* tests, *df* = 8, Site 1, *P*>0.09; Site 2, *P*>0.05). In one of the sites, however, there was greater variability among kelps on pilings with urchins than on reefs (Figure 2).

**Table 3:** Analyses of mean percentage covers of bryozoans on laminae of *E. radiata* for each individual in the experimental transplantation of urchins to pilings in 2007 (a) and 2006 (b).†

	Source	*df*	MS	*F*	*P*
a)	Site	1	1022	2.28	ns
	Treatment	1	7106	14.49	**
	Site×Treatment ^‡^	1	344		
	Residual	16	500		
b)	Site	1	1748	10.54	**
	Treatment	1	14027	9.52	ns
	Site×Treatment	1	1473	8.88	**
	Residual	16	166		
	2006, SNK: Site 1, P>TP **; Site 2, P>TP **				

†Site was a random factor with 2 levels and treatment was fixed with 2 levels (P, TP). The replicates were the kelps (*n* = 5). Cochran's test was used to test assumptions of homogeneity (2007, *C* = 0.53 ns; 2006, *C* = 0.46 ns). Non-significant interactions (^‡^) were pooled when ns with *P*>0.25. SNK tests of means were done where there were interactions. **, *P*<0.01; ns, *P*>0.05.

In 2006, there was a significant interaction between sites and treatments 1 month after urchins were experimentally transplanted to pilings (ANOVA, *F*_1,16_ = 8.88, *P*<0.01; Table 3). Nevertheless, there was a significantly smaller cover of bryozoans on kelps to which urchins had been experimentally transplanted (TP) than on undisturbed ones (P) for each site (SNK tests, *P*<0.01; Table 3). Mean covers of bryozoans on kelps on pilings to which urchins had been added (TP) remained significantly greater than on reefs (U) in each site (*t* tests, *df* = 8, *P*<0.01; Figure 2).

Procedural controls on reefs (translocated, TL; disturbed, D) did not differ from the undisturbed treatment (U; ANOVA, 2006: *F*_2,2_ = 3, *P*>0.25; 2007: *F*_2,2_ = 8, *P*>0.11).

## Discussion

In Sydney Harbour, pier-pilings influenced abundances of kelp epibiota directly due to the provision of shade and indirectly due to smaller densities of sea-urchins on pilings, supporting models 1 and 2.

One of the differences between pilings and reefs as habitats is that pilings support piers that shade the organisms that occur underneath. Shade increases recruitment of many sessile invertebrates [22] and has been shown to influence covers of sessile organisms on artificial structures [10], [30]. Shade directly influenced covers of bryozoans on laminae of kelps in this study, although this effect could have been partially due to differences in hydrodynamics. Control structures allowed controlling for potential artefacts caused by the structures used to shade kelps on reefs, but because mesh was used instead of a transparent cover to successfully allow light to penetrate, there may have been small differences in water-flow. Covers on shaded kelps on reefs did not, however, reach the covers on kelps on pilings. This suggests that other factors also affected covers of bryozoans.

Pilings also reduced abundances of the sea-urchin *Holopneustes purpurascens*, which, in turn, increased covers of invasive bryozoans on kelps. Although models that might explain the difference in densities of urchins between pilings and reefs were not examined here, one possible explanation may be differential predation. Man-made structures attract fishes [31], which may feed on urchins. Predation of adult urchins is, however, unlikely to explain the observed pattern because none of the urchins transplanted to pilings was lost during the experiment. Alternatively, recruitment of urchins may be affected by post-settlement mortality caused by fishes [32], [33]. There is also the possibility that abundances of urchins differ because of variations in their settlement [34]. In Sydney, settlement and metamorphosis of *H. purpurascens* occur on the red algae *Delisea pulchra* (where greater numbers of juveniles are found) [35] and not on its host as adults, *E. radiata* [36]. *D. pulchra* was never observed on pilings at the locations studied. Smaller abundances or absence of this red alga on pilings may therefore cause smaller recruitment of urchins to this habitat. Before drawing any firm conclusions, however, these models must be tested experimentally in the field. Understanding which factors and processes influence densities of urchins on pilings is of great importance if managers want these artificial structures to resemble natural reefs.

The next step would be to determine the mechanisms by which urchins affect bryozoans. Urchins may directly affect epibiota by predation. *H. purpurascens* can consume ∼1 g individual^−1^ of kelp per day [23]. Urchins may feed more on fouled algae than on those without epibiota [37]. If this was the case, epibionts may ‘interfere’ with grazing indirectly enhancing the consumption of kelps by urchins [38]. There are, however, other ways besides consumption in which they could affect bryozoans. For example, they could graze on laminae of kelps making them more likely to be torn apart by water-flow (e.g. currents and swell) thereby causing a decrease in abundances of epibiota. They could also displace new settlers whilst moving and/or feeding on the kelp [26], [27] or reduce the area available for settlement by folding the laminae. These and alternative models about the mechanisms by which urchins affect covers of bryozoans need to be tested experimentally.

The removal of urchins appeared to be more important than shade in explaining differences in covers between habitats; each explained approximately 50% and 30% of covers on pilings, respectively. It appears, therefore, that indirect effects of pilings (i.e. via urchins) have a greater influence in the abundance of epibiotic bryozoans than do the direct effects (i.e. through shade). Menge [39], for instance, showed that indirect effects accounted for approximately 40% of the changes in the structure of assemblages on a subset of intertidal rocky shores. In some locations, however, indirect effects explained more than 60% of the changes. Indirect effects from other types of artificial structures may also be more important influences on the structure of assemblages than are direct effects. Roads, for example, affect organisms directly by changing abiotic components of landscapes, such as light, temperature, wind, noise, etc. They also affect organisms indirectly, e.g. they can facilitate the introduction and spread of exotic species, which, in turn, can produce drastic changes in assemblages [5], [40].

The failure of covers of bryozoans in experimental patches to increase or decrease to match those on unmanipulated habitats of the opposite type could have been due to the experiments not lasting long enough. For instance, kelps on pilings have been shaded for their entire life, whilst kelps on reefs were shaded for one month, which might not have been sufficient (see e.g. [10]). This seems, however, unlikely. When kelps were transplanted from reefs to pilings and *vice versa* in previous experiments, covers became similar to those on the opposite habitat after 1 month [15]. Thus, 1 month was considered here to be the appropriate time to measure the predictive changes. Alternatively, this could be explained by the timing of the experiment, e.g. recruitment may have been small at that time of the year. In Sydney Harbour, bryozoans seem to grow fast and recruit in large numbers during the months the experiment was done [28], so this explanation seems unlikely.

The points considered previously indicate the necessity of going beyond the study of ecological patterns to investigate the processes that determine them because this information is crucial to develop successful strategies for management and conservation [41]. There are direct influences due to the provision of shade by the modified habitat and indirect influences due to smaller densities of sea-urchins on modified habitats. The rapid increase of the human population globally [42] and some of the consequences of climatic change, e.g. increases of sea-level, frequency and intensity of storms, etc. [43], [44], may increase current rates of modification of shorelines. It is therefore necessary to increase our understanding of the effects of these structures on ecological patterns and processes to provide information and practical advice for conservation and management. This information can be used practically to minimise the impacts of artificial habitats. Here, reducing the shade caused by piers and transplanting urchins to pilings may minimize the changes in biodiversity caused by pilings. Altering the design of built structures to be better mimics of natural habitats will contribute to the conservation of local biodiversity by preserving natural patterns of abundances and distribution of organisms and the processes that determine them. Further, this can mitigate other potential adverse effects of anthropogenic modification of habitats, e.g. by reducing the invasibility of natural systems and increasing their resilience and stability [45]. In this study, differences in covers between pilings and reefs appeared to be mostly dependent on one non-indigenous species of bryozoan: *Membranipora membranacea*. Fouling by these organisms can have negative effects on the kelps, e.g. increased grazing due to epibiota [37], reductions in the area of alga for photosynthesis [18] and increasing drag [46], among others [47], which may, in turn, affect organisms that use kelps as a resource. Identifying the mechanisms by which modified habitats influence invaders is therefore crucial to provide sound information for management.

The issues considered in this paper about direct and indirect effects of human modification of habitat are also relevant to other types of alteration of habitats. Natural disturbances modify underlying habitats, having direct and/or indirect effects on the organisms thereby influencing the structure of assemblages [26], [48]. Although direct effects of natural disturbances have been extensively reported, many potential indirect effects are still poorly understood. Manipulative experiments in the field designed to separate between these types of effects not only provide valuable information to mitigate human impacts, but also increase our understanding of the ecology of natural assemblages.
